# Stakeholder Perspectives on Reducing Hospitalizations Among Nursing Home Residents

**DOI:** 10.1093/geroni/igaa057.280

**Published:** 2020-12-16

**Authors:** Denise Tyler, Cleanthe Kordomenos, Melvin Ingber

**Affiliations:** 1 RTI International, Waltham, Massachusetts, United States; 2 RTI International, Research Triangle Park, North Carolina, United States; 3 RTI International, Rockville, Maryland, United States

## Abstract

Organizations in seven states have been participating in the Center for Medicare and Medicaid Innovation (CMMI) initiative aimed at reducing potentially avoidable hospitalizations among long-stay nursing home (NH) residents. The purpose of this study was to identify market and policy factors that may have affected the initiative in those states. Forty-seven interviews were conducted with key stakeholders in the seven states (e.g., representatives from state departments of health, state Medicaid offices, and nursing, hospital and nursing home associations) and qualitatively analyzed to identify themes across states. Few policies or programs were found that may have affected the initiative; only New York (NY) was found to have state policies or programs specifically aimed at reducing hospitalizations. Market pressures reported in most states were similar. For example, stakeholders reported that the increased availability of home and community-based services and the growing presence of managed care are contributing to higher acuity among both long and short stay residents and that reimbursement rates and staffing have not kept up. Stakeholders suggested greater presence of physicians and nurse practitioners in NHs, better training around behavioral health issues for frontline staff, and more advance care planning and education of families about end of life may help further reduce NH hospitalizations. We also found that all states, except NY, had regional coalitions of health care related organizations focused on improving some aspect of care, such as reducing hospital readmissions. These coalitions may suggest ways that organizations can work together to reduce hospitalizations among NH residents.

